# The Antisaccade Task: Visual Distractors Elicit a Location-Independent Planning ‘Cost’

**DOI:** 10.1371/journal.pone.0122345

**Published:** 2015-04-01

**Authors:** Jesse C. DeSimone, Stefan Everling, Matthew Heath

**Affiliations:** 1 School of Kinesiology, University of Western Ontario, London, ON, Canada; 2 Department of Physiology and Pharmacology, Department of Psychology, Robarts Research Institute, and Graduate Program in Neuroscience, University of Western Ontario, London, ON, Canada; 3 School of Kinesiology and Graduate Program in Neuroscience, University of Western Ontario, London, ON, Canada; University of Leicester, UNITED KINGDOM

## Abstract

The presentation of a remote – but not proximal – distractor concurrent with target onset increases prosaccade reaction times (RT) (i.e., the *remote distractor effect*: RDE). The competitive integration model asserts that the RDE represents the time required to resolve the conflict for a common saccade threshold between target- and distractor-related saccade generating commands in the superior colliculus. To our knowledge however, no previous research has examined whether remote and proximal distractors differentially influence antisaccade RTs. This represents a notable question because antisaccades require decoupling of the spatial relations between stimulus and response (SR) and therefore provide a basis for determining whether the sensory- and/or motor-related features of a distractor influence response planning. Participants completed pro- and antisaccades in a target-only condition and conditions wherein the target was concurrently presented with a proximal or remote distractor. As expected, prosaccade RTs elicited a reliable RDE. In contrast, antisaccade RTs were increased independent of the distractor’s spatial location and the magnitude of the effect was comparable across each distractor location. Thus, distractor-related antisaccade RT costs are not accounted for by a competitive integration between conflicting saccade generating commands. Instead, we propose that a visual distractor increases uncertainty related to the evocation of the response-selection rule necessary for decoupling SR relations.

## Introduction

**Fig 1:**
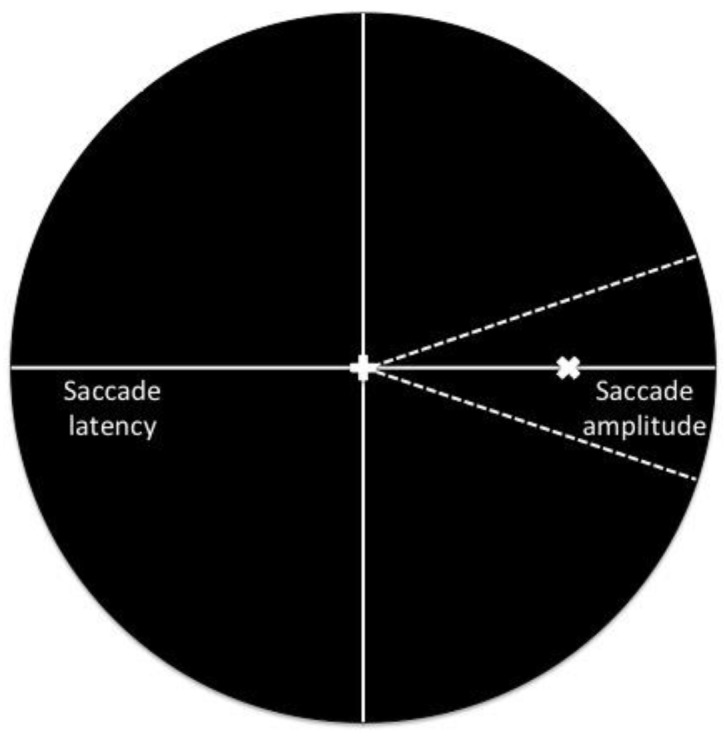
Figure adapted from Walker et al. [2] illustrating the converse effects of proximal and remote distractors on saccade amplitude and reaction time. Distractors presented ipsilateral and within ±20° from the horizontal target (“x”) axis (i.e., the area contained within the hatched lines) bias saccade amplitudes toward the distractor (i.e., the *global effect*) but do not influence reaction time, whereas distractors located outside of this critical window (i.e., > 20° from the horizontal target axis or at the location of central fixation) elicit a delay in reaction time (i.e., the *remote distractor effect*) but do not influence saccade amplitude.

Prosaccades are rapid eye movements that bring a target of interest onto the fovea. The majority of work involving prosaccades has employed an experimental paradigm wherein a target stimulus is presented in an impoverished (i.e., empty) visual environment. Results from this work have shown that prosaccades are characterized by short latencies and accurate endpoints—a finding attributed to their mediation via dedicated retinotopic motor maps in intermediate layers of the superior colliculus (SC) [1]. It is, however, important to recognize that the visual environments in which humans interact are rarely comprised of a single stimulus; rather, successful prosaccades require disentangling the location of a target from task-irrelevant visual cues. As an experimental corollary, the visual distractor paradigm requires that participants ignore the presentation of a task-irrelevant visual distractor and saccade to a visual target. A number of studies have shown that the location of a distractor relative to a target differentially influences prosaccade reaction times (RT) and amplitudes. For example, Walker and colleagues [2] (see also [3]) reported that the concurrent presentation of a target and *remote distractor* (i.e., > 20° in angular coordinates from the target axis) produced longer RTs than when the target was presented alone or when presented with a *proximal distractor* (i.e., within ± 20° of the ipsilateral target axis) (*the remote distractor effect*: RDE) (see Fig 1 for schematic representation). In turn, distractor location elicits a converse effect on prosaccade amplitudes such that proximal distractors bias amplitudes toward the distractor (i.e., the *global effect*), whereas remote distractors do not influence amplitudes [2, 4–6] (for review see [7]).

A number of studies have attributed the RDE and global effect to the motor-related properties of neurons in the SC. In particular, the *competitive integration model* (CIM) [8, 9] contends that the visual information supporting target and distractor are concurrently transformed into motor programs within a common retinotopic motor map in the intermediate layers of the SC. Given the common retinotopic mapping, target- and distractor-specific saccade neurons compete for a common threshold and create conflicting saccade generation commands that require additional time to resolve (see also [10]). More directly, the CIM asserts that the RDE results from a long-range intercollicular inhibitory pathway [11] in which saccade-related activity at one location inhibits the activation of distant locations within the motor map. Thus, active saccade neurons associated with a remote distractor delay the motor-related buildup properties serving a saccade to the target location [9] (for alternative account see [2, 12]). In turn, when a distractor is presented proximal to a target the motor activity related to each stimulus merges into a single movement vector that represents a spatially averaged response [13, 14]. Notably, although the spatially averaged response of a proximal distractor does not engender a cost to saccade latency it does result in a response that falls between the target and distractor (i.e., the global effect).

To our knowledge, previous work has not examined location-specific distractor effects for antisaccade planning times. In particular, antisaccades represent a non-standard motor task requiring that participants saccade mirror-symmetrical (i.e., 180° spatial transformation) to the location of an exogenously presented target. Extensive evidence has shown that antisaccades produce longer RTs [15, 16], increased directional errors [17], and less accurate and more variable endpoints [18–20] than prosaccades. Moreover, human and non-human primate neuroimaging and electrophysiological findings have attributed the antisaccade planning ‘cost’ to a two-component process requiring the top-down inhibition of a stimulus-driven prosaccade (i.e., response suppression) and the *visual* remapping of a target to a mirror-symmetrical location in space (i.e., vector inversion) (for review see [21]).

**Fig 2:**
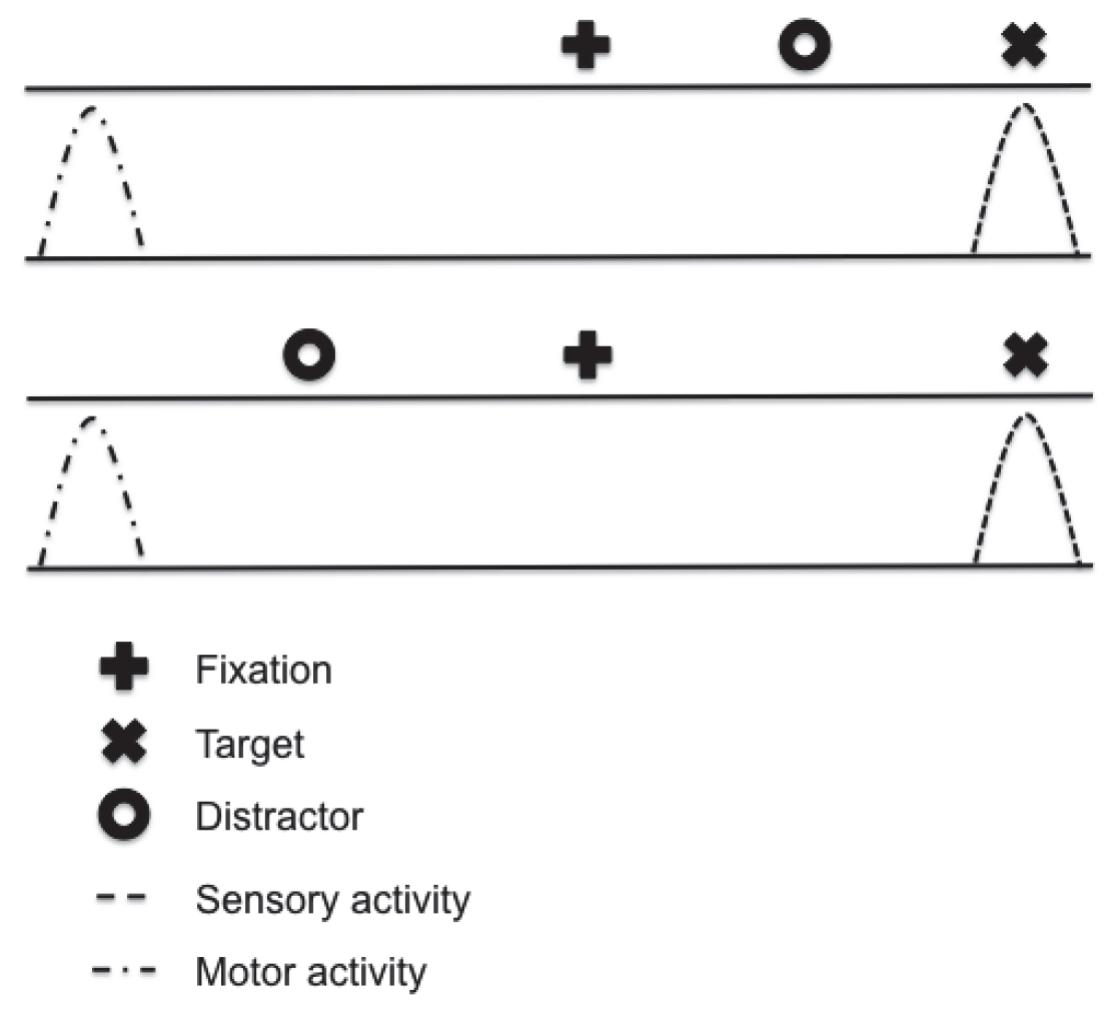
Sensory- and motor-related spatial properties of a distractor in the antisaccade task. The top panel shows that although the sensory-related activity of a ‘proximal’ distractor in an antisaccade task is in the same visual field as the target stimulus, the motor-related activity is ‘remote’ (i.e., the opposite visual field). The bottom panel shows that the sensory-related activity of a ‘remote’ distractor in an antisaccade task is in the visual field opposite to the target (i.e., it is remote); however, the motor-related activity is proximal to the target (i.e., the same visual field).

The present investigation sought to determine whether distractor location influences antisaccade planning times in a manner similar to prosaccades. Notably, the decoupled stimulus and response (SR) relations associated with the antisaccade task provide a basis for determining whether the sensory (i.e., target)- and/or motor (i.e., goal)-related features of a distractor influence response planning. In order to highlight this issue, Fig 2 shows that in an antisaccade task the sensory properties (i.e., veridical location) of a ‘proximal’ distractor are contained within the same visual field as the target stimulus; however, the goal-location of the response is in the mirror-symmetrical visual field (i.e., remote to the target’s veridical location). In other words, the sensory-related property of the distractor is proximal to the target, whereas the motor-related property of the distractor is remote to the target. In turn, Fig 2 shows the converse relationship associated with a ‘remote’ distractor. As such, a corollary prediction drawn from the CIM regarding antisaccades is that the saccade-related buildup properties serving a proximal—but not a remote—distractor should delay planning times. Indeed, a location-specific increase in RT for a proximal distractor would support the contention that distractor costs—in the antisaccade task—arise from a motor-related competition between conflicting and directionally alternative saccade generation commands. More directly, a proximal distractor would result in distractor and saccade-related motor activity that is encoded within remote areas of the retinotopic motor maps of the SC. As such, a proximal—but not remote—distractor would induce a long-range attenuation of motor-related buildup neurons serving antisaccade planning. As an alternative to the RDE, it is possible that the top-down response-selection rule necessary for a response with decoupled SR relations influences planning times independent of the distractor’s spatial location. The basis for this prediction stems from a choice-RT study by Kveraga and colleagues [22] showing that increasing the number of SR alternatives (i.e., distractors) associated with an antisaccade—but not prosaccade—task conforms to the log-linear increase in RT defined by *Hick’s law* [23]. According to Kveraga et al., antisaccades conform to Hick’s law because the top-down requirements of SR decoupling requires: (1) an obligatory response-selection strategy that entails the spatial transformation of the target vector, and (2) an increased response-selection uncertainty related to the processing of each potential SR alternative. In turn, Kveraga et al. proposed that prosaccades do not adhere to Hick’s law because localization of the target among distractor(s) serves as the imperative to automatically map the target’s spatially encoded visual activity into a motor response. As such, if response-selection uncertainty underlies antisaccade distractor costs then RT delays should be independent of the distractor’s spatial location.

**Fig 3:**
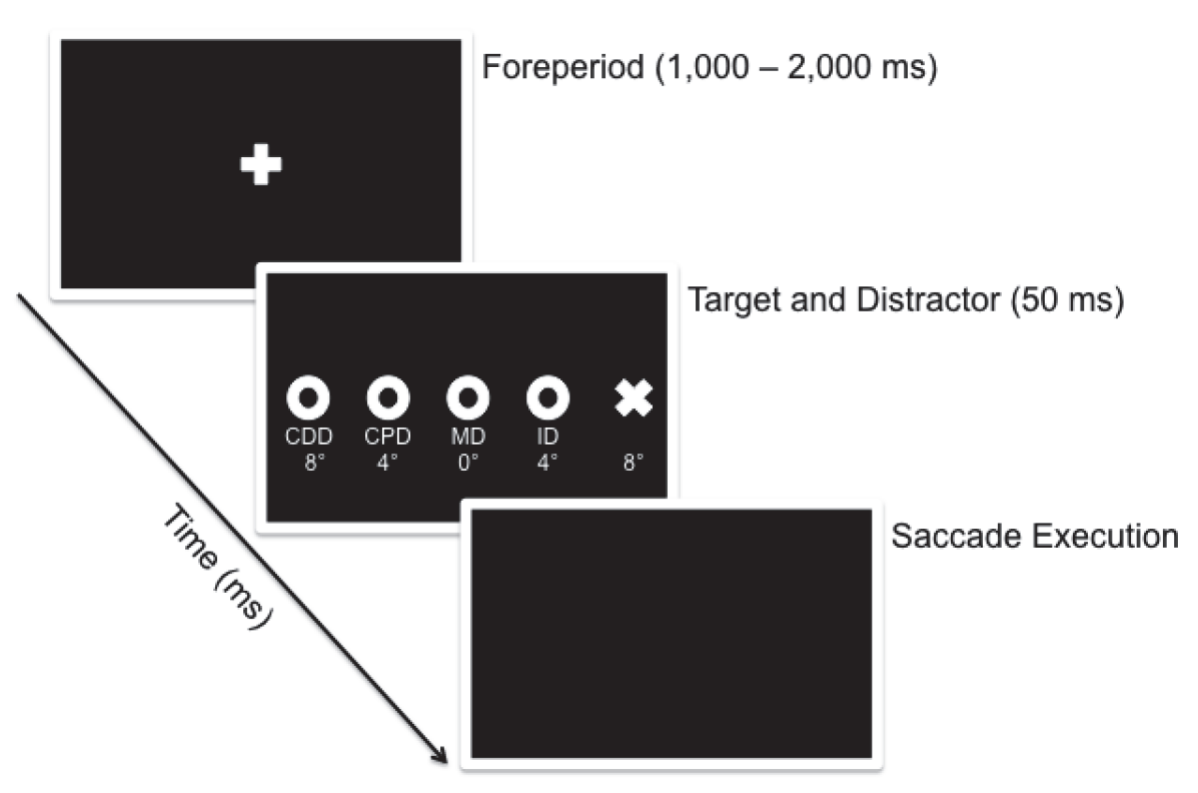
Timeline of visual events for a target presented in the right visual field. A white fixation cross was presented for a randomized foreperiod (1,000–2,000 ms). Following the foreperiod, the fixation cross was extinguished and a visual target was presented left or right of fixation for 50 ms. For 80% of trials, a visual distractor was presented concurrent with the target at a proximal (i.e., ipsilateral distractor: ID), or remote (MD, CPD, CDD) spatial location along the horizontal target axis. For the remaining trials a target was presented without a distractor (TO). The onset of the target (and distractor) served as the imperative to complete the instructed pro- or antisaccade.

The present study sought to determine whether—and to what degree—the spatial location of a distractor relative to a visual target differentially influences pro- and antisaccade planning times. To that end, we employed target and distractor locations similar to that used in Walker et al.’s [2] examination of the RDE for prosaccades (see Experiment 1a of that work). In particular, pro- and antisaccades were completed in a condition wherein a target was presented alone (i.e., target-only condition) and conditions wherein a distractor was presented at proximal and remote locations to the target and along the same horizontal axis (see Fig 3). In terms of potential research outcomes, if antisaccades are susceptible to the same long-range inhibition as prosaccades than the motor- and not the sensory-related location of the distractor should result in an increase in RT. In turn, if an obligatory process of response-selection influences antisaccade planning then distractor-related RT costs should be location-independent.

## Methods

### Participants

Fifteen individuals (11 female and 4 male: age range 18–30 years) from the University of Western Ontario community volunteered for this experiment. All participants were self-declared right-hand dominant with normal or corrected-to-normal vision. Participants signed consent forms approved by the Office of Research Ethics, the University of Western Ontario, and this work was conducted in accord with the ethical standards outlined in the Declaration of Helsinki.

### Apparatus and procedure

Participants were seated at a table (775 mm in height) with their head placed in a head-chin rest for the duration of the experiment. Visual stimuli were presented on a 30-inch LCD monitor (60 Hz, 8 ms response rate, 1280 × 960 pixels, Dell 3007WFP, Round Rock, TX, USA) placed 550 mm from the participant and centered on their midline. Point of gaze data were obtained from each participant’s left eye via a video-based eye-tracking system (Eye-Trac 6: Applied Science Laboratories, Bedford, MA, USA) sampling at 360 Hz. Prior to data collection a nine-point calibration of the viewing space was performed and confirmed via an immediate follow-up calibration. Two additional monitors that were visible only to the experimenter provided: (1) real-time point of gaze information, (2) a visual depiction of trial-to-trial saccade kinematics (i.e., displacement, velocity), and (3) information on the accuracy of the eye tracking system (i.e., to allow for drift correction or re-calibration when necessary). All computer events were controlled via MATLAB (Version 7.8.0, The MathWorks, Inc., Natick, MA, USA) and the Psychophysics Toolbox extensions (version 3.0) [24]. The lights in the experimental suite were extinguished during data collection.

Visual stimuli included a white fixation cross (0.7°) centered horizontally on the monitor and at the eye level of the participant. White diagonal crosses (0.7°) served as target stimuli and were located 8° left and right of fixation. Additionally, unfilled white circles (0.7°) served as task-irrelevant stimuli (i.e., distractors) and were presented along the same horizontal axis as the fixation and target stimuli. Distractors were located: (1) ipsilateral to the target at an eccentricity of 4° from the fixation cross (i.e., ipsilateral distractor: ID), (2) at the location of the fixation cross (i.e., 0° and henceforth referred to as the midline distractor: MD), (3) contralateral to the target at an eccentricity of 4° from the fixation cross (i.e., contralateral proximal distractor: CPD), and (4) contralateral to the target and at an eccentricity of 8° from fixation cross (i.e., contralateral distal distractor: CDD) (see Fig 3). The different distractor locations were selected to be comparable to those employed in Experiment 1a of Walker et al’s [2] examination of the RDE for prosaccades.

At the start of each trial, the fixation cross was presented and participants were instructed to direct their gaze to its location. Once a stable gaze of the fixation cross was achieved (±1.5° for 420 ms), a randomized foreperiod (1,000–2,000 ms) was initiated during which time the fixation cross remained visible. Following the foreperiod, the fixation cross was extinguished and a target stimulus (i.e., target-only condition: TO), or target stimulus with distractor (i.e., ID, MD, CPD, CDD conditions) was presented for 50 ms (see Fig 3 for timeline of visual events). The onset of the target stimulus served as the cue to generate a pro- or antisaccade “as quickly and accurately as possible” and to ignore the irrelevant distractor when present. Prosaccades entailed a response to the target’s veridical location, whereas antisaccades entailed a response mirror-symmetrical to the veridical target location. A 50 ms target (and distractor) presentation was used so that the target was unavailable throughout response planning and execution—a method requiring visual vector inversion for antisaccades as opposed to a continuous target presentation wherein antisaccades may be mediated via an obligatory shift of attention from the target to a homologous region in space [25]. Further, the brief presentation ensured that neither pro- nor antisaccades trials provided retinal feedback related to response accuracy.

Participants completed a single block of prosaccades and a single block of antisaccades and the ordering of blocks was randomized. Pro- and antisaccades were performed in separate blocks because randomly interleaving tasks inhibits the normally stimulus-driven nature of prosaccades and results in planning behaviours that are akin to antisaccades [25, 26]. In particular, an antisaccade completed prior to a prosaccade engenders a lingering non-standard task-set that selectively increases prosaccade RTs (i.e., the unidirectional prosaccade switch cost [26, 27]). As such, the block ordering technique used here isolated distractor location effects separately for pro- and antisaccades. As noted above, responses were completed in target-only (TO) and four distractor conditions (ID, MD, CPD, CDD) that were randomly interleaved within each block. In addition, the visual field (left, right) associated with the target stimulus was randomized within each block. For each block, participants completed 12 trials to each of the aforementioned trial-type by visual field combinations (i.e., 240 total experimental trials).

### Data analysis and dependent variables

Displacement data were filtered offline using a dual-pass Butterworth filter employing a low-pass cut-off frequency of 15 Hz. Filtered displacement data were used to compute instantaneous velocities via a five-point central finite difference algorithm. Acceleration data were similarly obtained from the velocity profiles. Saccade onset was determined on the basis of velocity and acceleration values that exceeded 30°/s and 8,000°/s^2^, respectively. Saccade offset was marked when velocity fell below 30°/s for 15 consecutive frames (i.e., 42 ms). The dependent variables were reaction time (RT: time from target onset to movement onset) and saccade amplitude in the horizontal movement direction. Dependent variables were examined via 2 (task: prosaccade, antisaccade) by 5 (trial-type: TO, ID, MD, CPD, CDD) repeated measures ANOVAs. Post-hoc decompositions for trial-type were completed by contrasting each distractor condition to their respective pro- or antisaccade TO condition counterpart via paired samples t-tests. Only directionally correct pro- and antisaccade trials were analyzed. Accordingly, for each participant we excluded on average 3% and 10% of pro- and antisaccade trials, respectively. In particular, prosaccade errors were elicited only when a distractor was presented contralateral to the target and errors for CPD (11%) and CDD (7%) conditions did not reliably differ, *t*(14) = 1.42, *p* = 0.17. For antisaccades, the MD condition (5%) elicited fewer errors than the TO condition (16%), *t*(14) = -2.75, *p* = 0.02, whereas the errors for the ID (15%), CPD (8%), and CDD (10%) conditions did not reliably differ from the TO condition (*t*s(14) < 1). We further note that for each participant an average of 2% of trials were removed due to: (1) signal loss (i.e., blinking), (2) RT greater than two standard deviations above the mean group performance (i.e., RT > 700 ms), and (3) an anticipatory response (i.e., RT < 85 ms).

## Results

### Reaction time

**Fig 4:**
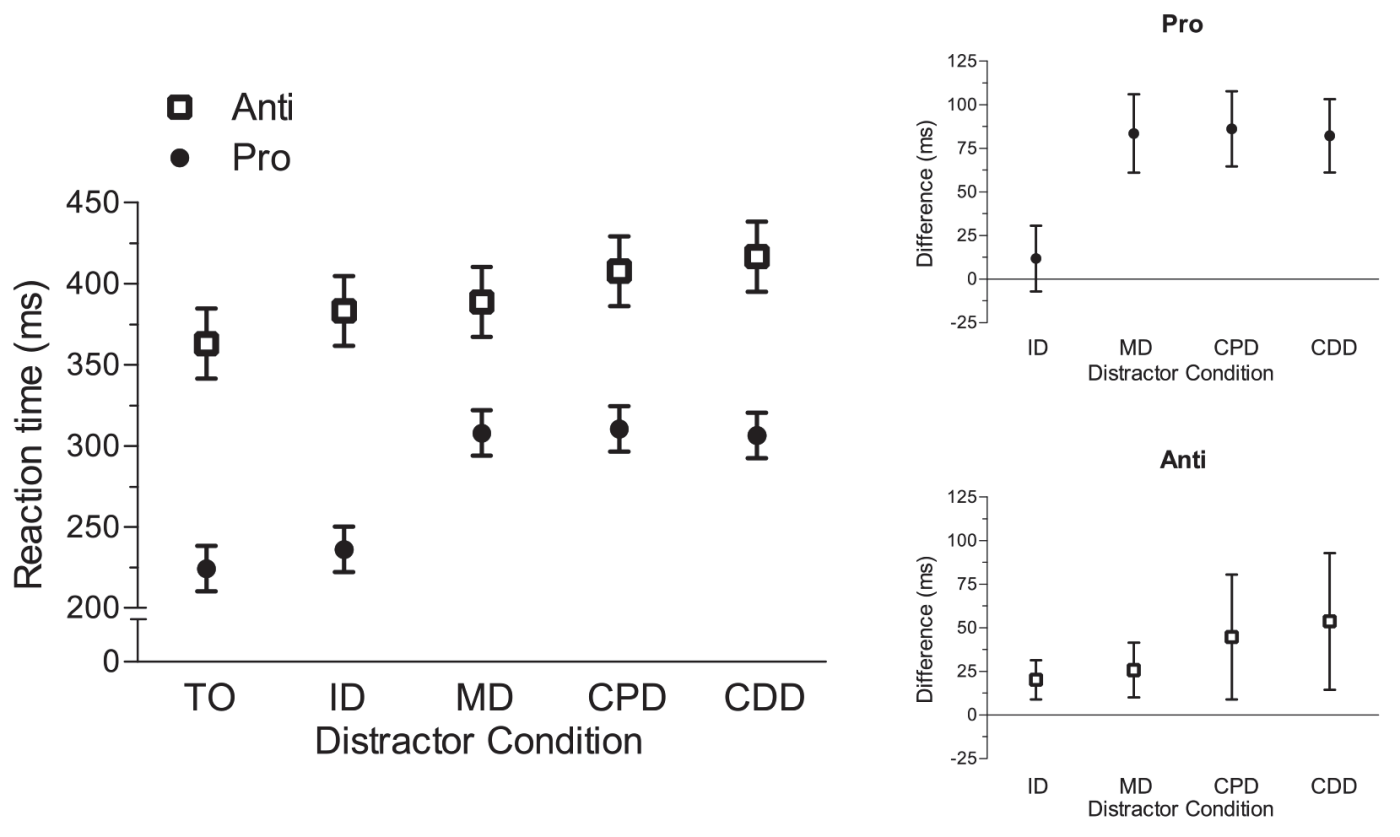
The main panel depicts mean reaction time (ms) for pro- (i.e., closed circles) and antisaccades (i.e., open squares) in target-only (TO), proximal distractor (ID), and remote (MD, CPD, CDD) distractor conditions. Error bars for this panel represent within-participant 95% confidence intervals [28]. Confidence intervals were computed based on the mean-squared error term for trial-type separately for pro- and antisaccades. The top-right and bottom-right offset panels show mean distractor RT difference scores for pro- and antisaccades, respectively. Error bars represent between-participant 95% confidence intervals [29, 30]. The absence of overlap between error bars and zero (i.e., horizontal axis) provides a graphical depiction of a reliable difference that can be interpreted inclusive to a test of the null hypothesis.

Results yielded main effects of task, *F*(1,14) = 70.80, *p* < 0.001, trial-type, *F*(4,56) = 25.12, *p* < 0.001, and their interaction, *F*(4,56) = 5.64, *p* < 0.002. As expected, Fig 4 shows that prosaccades (277 ms, SD = 37) had shorter RTs than antisaccades (391 ms, SD = 72). Moreover, Fig 4 shows that RTs for prosaccades in the remote distractor conditions (i.e., MD, CPD, CDD) were longer than their TO condition counterpart, *t*s(14) > 7.97, *p*s < 0.001, whereas the ID and TO conditions did not reliably differ, *t*(14) = 1.35, *p* = 0.20. For antisaccades, ID, MD, CPD and CDD conditions produced longer RTs than the TO condition, *t*s(14) > 2.68, *p*s < 0.02. Thus, prosaccades elicited a RDE, whereas antisaccade RTs were increased independent of the distractor’s spatial location.

Although the preceding analyses provided a basis to determine which distractor conditions reliably differed from the TO condition, it does not provide a framework to identify within-task differences in the *magnitude* of distractor costs. Thus, we computed reaction time difference scores (i.e., distractor condition minus TO condition) separately for each task (i.e., pro- and antisaccade) and distractor condition combination. Pro- and antisaccade difference scores were submitted to separate one-way ANOVAs. As expected, results for prosaccades showed that the ID condition (12 ms, SD = 34) produced a smaller difference score than the other distractor conditions (MD: 84 ms; SD = 40, CPD: 86 ms; SD = 39, and CDD: 82 ms; SD = 38), *F*(3,42) = 33.04, *p* < 0.001; however, MD, CPD and CDD difference scores did not reliably differ, *F*(2,28) = 0.25, *p* = 0.78 (see Fig 4). For antisaccades, the magnitude of the distractor cost did not reliably differ across conditions (ID: 20 ms; SD = 20, MD: 25 ms; SD = 28, CPD: 45 ms; SD = 64, and CDD: 54 ms; SD = 70), *F*(3,42) = 2.22, *p* = 0.10 (see Fig 4).

### Saccade amplitude

**Fig 5:**
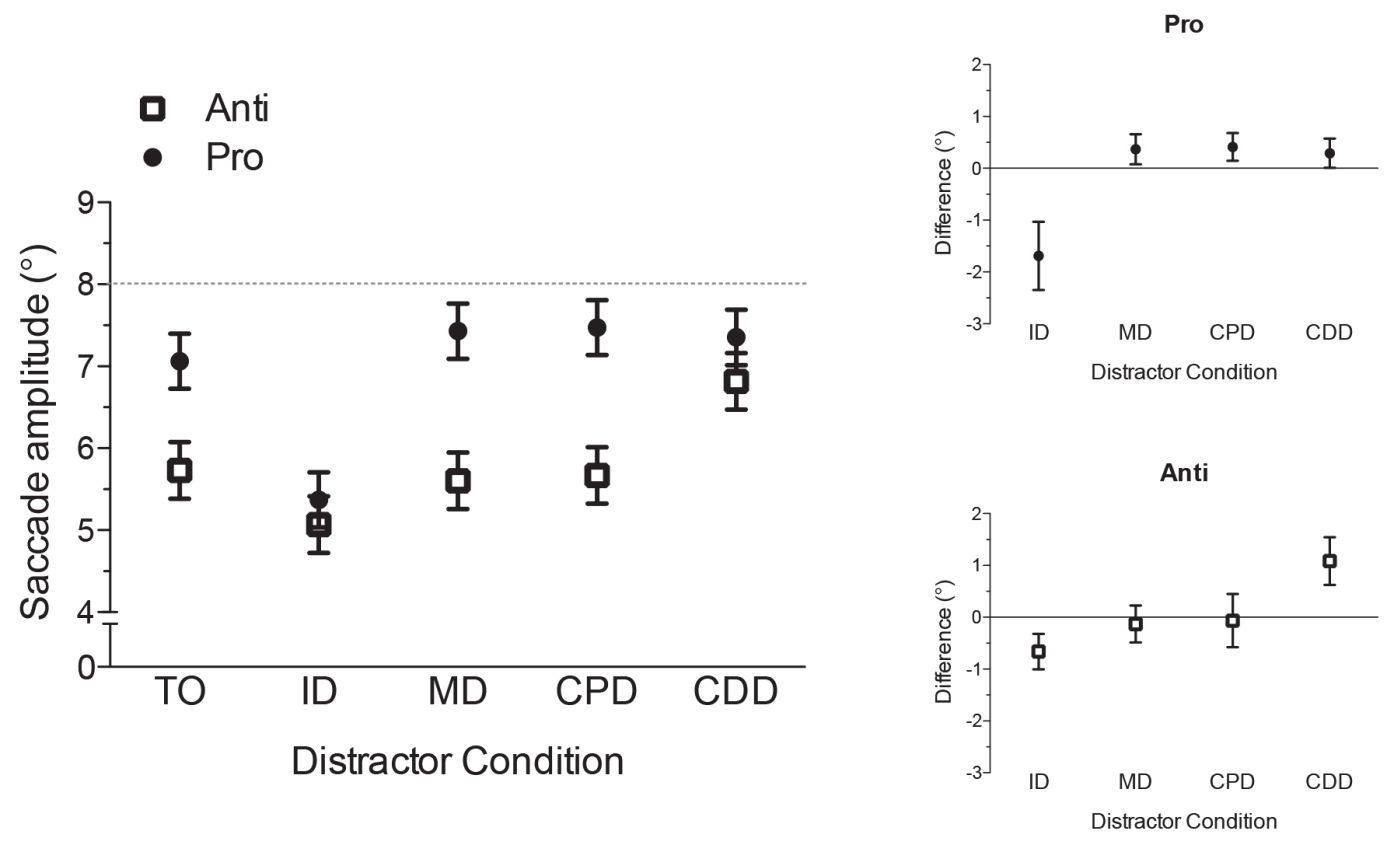
The main panel depicts mean saccade amplitude (°) in the horizontal direction for pro- (i.e., closed circles) and antisaccades (i.e., open squares) completed in target-only (TO), proximal distractor (ID), and remote distractor (MD, CPD, CDD) conditions. Error bars for this panel represent within-participant 95% confidence intervals [28]. The top-right and bottom-right offset panels show mean distractor amplitude difference scores for pro- and antisaccades, respectively. Error bars represent between-participant 95% confidence intervals [29, 30]. The absence of overlap between error bars and zero (i.e., horizontal axis) provides a graphical depiction of a reliable difference that can be interpreted inclusive to a test of the null hypothesis.

Saccade amplitude data yielded main effects of task, *F*(1,14) = 18.01, *p* < 0.002, trial-type, *F*(4,56) = 38.32, *p* < 0.001, and their interaction, *F*(4,56) = 9.82, *p* < 0.001. Recall that veridical target location was 8°. Thus, and as shown in Fig 5, pro- (6.9°, SD = 0.9) and antisaccades (5.8°, SD = 1.5) elicited an undershooting bias; however, the magnitude of the bias was larger for the latter task. As well, Fig 5 shows that prosaccade amplitudes in the ID condition were less than the TO condition, *t*(14) = -5.51, *p* < 0.001, whereas MD, CPD, and CDD conditions were greater than the TO condition, *t*s(14) > 2.23, *p*s < 0.05. For antisaccades, ID and CDD conditions produced amplitudes that were less than and greater than the TO condition, respectively, *t*s(14) = -4.14 and 5.04, *p*s < 0.002. In turn, amplitudes MD and CPD conditions did not reliably differ from the TO condition (*t*s(14) < 1).

To determine if trial-type elicited a planning-related speed-accuracy trade-off [31] we computed correlation coefficients relating participant-specific mean RT and amplitude data separately for each pro- and antisaccade trial-type (i.e., TO, ID, MD, CPD, CDD). Correlation coefficients were then subjected to a Fisher r-to-z transformation and submitted to 2 (task: prosaccade, antisaccade) by 5 (trial-type: TO, ID, MD, CPD, CDD) repeated measures ANOVA. Results yielded a significant task by distractor interaction, *F*(4,56) = 5.04, *p* < 0.005. In terms of prosaccades, post hoc examination revealed a larger RT-amplitude relation in the ID (0.56) compared to the TO condition (0.20), *t*(14) = 3.27, *p* < 0.01, whereas the MD (-0.01), CPD (-0.01), and CDD (0.24) conditions did not reliably differ from the TO condition, (*t*s(14) = -1.7, -1.7, and 0.21, respectively, *ps* > 0.11). In terms of antisaccades, the RT-amplitude relation for the ID condition (-0.20) was smaller than the TO condition (-0.02), *t*(14) = -2.20, *p* < 0.05, whereas the MD (-0.03), CPD (-0.06) and CDD (-0.06) conditions did not reliably differ from the TO condition (*t*s(14) < 1). Thus, the relationship between RT and amplitude was neither reliably nor consistently modulated across the different pro- and antisaccade trial-types used here.

## Discussion

### Prosaccade RTs: A replication of Walker et al. (1997)

The target and distractor conditions employed here included a subset of those used by Walker et al. [2]. Fig 4 shows that remote distractor conditions (i.e., MD, CPD, CDD) elicited longer prosaccade RTs than the TO condition, whereas the proximal distractor (i.e., ID) and TO conditions did not reliably differ. Moreover, the average cost of a remote distractor was 84 ms, and the magnitude of this effect did not vary across the different remote distractor locations. Thus, our results demonstrate a reliable RDE in line with Walker et al. Of course, such a finding is consistent with the CIM’s assertion that remote distractors delay prosaccade planning times via a long-range attenuation of preparatory activity in the SC. Notably, however, the RDE magnitude in the current experiment was larger than that reported in Walker et al’s original examination. In their work, contralateral and midline distractors elicited a mean RDE of 10 and 40 ms, respectively (see also [32]). To account for the discrepant findings, we note that the visual field (left, right) associated with the target stimulus in the current experiment was randomized within each block of trials, whereas Walker et al. restricted target presentation to a single visual field. As such, the use of a blocked target direction may have facilitated enhanced preparatory saccade-related activity in the SC corresponding to the target direction [33] and thereby improved discrimination of target and distractor at response cuing. Regardless of this difference, both studies reliably indicate that proximal and remote distractors differentially influence prosaccade planning times.

### Prosaccade amplitudes are influenced by proximal and remote distractors

**Fig 6:**
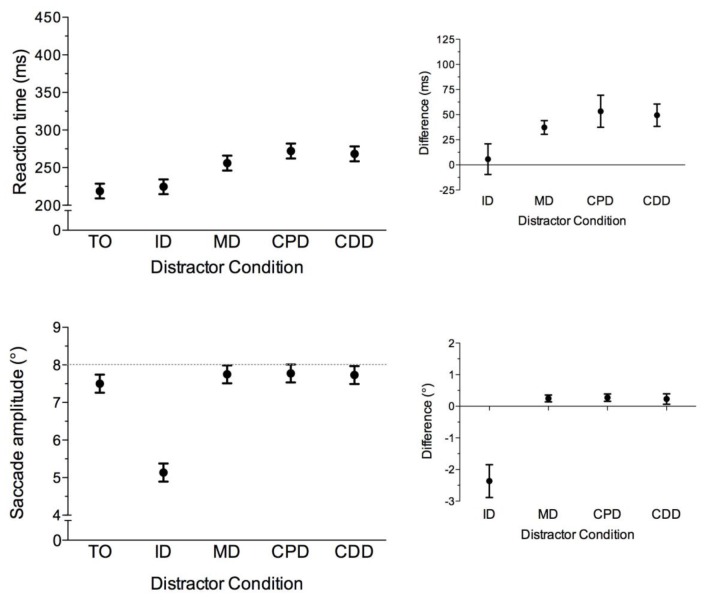
The main panels depict mean reaction time (ms) (i.e., upper panel) and saccade amplitude (°) (i.e., lower panel) for the supplemental experiment wherein prosaccades across TO, ID, MD, CPD, CDD trial-types were completed with target- or target and distractor vision throughout response execution. Results for RT showed that TO and ID conditions produced equivalent RTs, *t*(9) = 0.85, *p* = 0.42, whereas RTs for MD, CPD, and CDD conditions were greater than the TO condition, *t*s(9) > 7.57, *p*s < 0.001. Error bars for these panels represent within-participant 95% confidence intervals [28] and were computed based on the mean-squared error term for trial-type. The right offset panels show the mean reaction time and saccade amplitude difference scores. Error bars represent between-participant 95% confidence intervals [29, 30]. The absence of overlap between error bars and zero (i.e., horizontal axis) provides a graphical depiction of a reliable difference that can be interpreted inclusive to a test of the null hypothesis. Further, we note that Benson [37] employed a RDE paradigm wherein target and distractor vision was available throughout response execution and the target direction was randomized between left and right visual space. In contrast to Walker et al. [2], Benson reported a larger RDE magnitude for contralateral—compared to central—distractors. In line with Benson, prosaccade RT difference scores associated with the remote distractor conditions in the supplemental experiment yielded a significant main effect, *F*(1,9) = 7.09, *p* < 0.01, such that the contralateral distractors (i.e., CPD: 53 ms; *SD* = 22, CPD: 49 ms; *SD* = 15) produced a larger magnitude RDE than the midline distractor (i.e, MD condition: 37 ms; *SD* = 9), *t*s(9) > 2.79, *p*s < 0.05.

Prosaccades in the ID condition landed between the target and distractor. This result reported previously by Walker et al. [2] and others [4–6, 34] has been interpreted to reflect that the motor representation of target and distractor locations on a common short-range motor map within the intermediate layers of the SC results in the spatial averaging of motor-related saccade activity (i.e., the global effect). Notably, however, we also observed that amplitudes for remote distractor conditions were longer than the TO condition. This finding counters Walker et al. who reported a null amplitude effect of remote distractors. One possible explanation for the between-experiment discrepancy is that the current study employed a brief (50 ms) target (and distractor) presentation, whereas the target (and distractor) used in Walker et al.’s study was available throughout response execution. As such, the continuous target (and distractor) vision associated with Walker et al. may have served to support on- or offline corrections to the primary saccade trajectory [20, 35, 36]. To address this issue, we completed a supplemental experiment involving 10 participants (7 female and 3 male: age range 18–30 years) and used the same procedures as our main experiment with the only difference being that the target (and distractor) was available throughout response execution (i.e., target and distractor were extinguished at saccade offset). The results for the supplemental experiment are presented in Fig 6 and provide a replication of our main experiment. First, results for RT produced a reliable RDE in line with our main experiment. Second, amplitudes in the ID condition were more hypometric than the TO condition, *t*(9) = -10.32, *p* < 0.001, whereas amplitudes in the MD, CPD, and CDD conditions were less hypometric than the TO condition, *t*s(9) > 3.17, *p*s < 0.02. Thus, target and distractor vision throughout response execution does not account for the discrepancy between our results and Walker et al. As an alternative account, we note that Walker et al. employed six participants, whereas the main and supplemental experiments used here employed 15 and 10 participants, respectively. It is therefore possible that the detection of remote distractor effects may relate to statistical power. In addressing this issue we created 20 unique and pseudo-randomly sampled data sets involving six participants from each of our main and supplemental experiments and contrasted TO and CDD condition amplitudes via paired-samples t-tests. Results showed that that the probability of statistically equivalent amplitudes for TO and CDD conditions were 100% (20/20) and 60% (12/20) for the main and supplemental experiments, respectively. Given these findings, we propose that the null remote distractor effect reported in previous work may relate to an exiguous replication sample size.

In explaining the longer amplitudes in the remote distractor conditions we note that previous work has shown that manual and saccade trajectories ‘curve’ away from the location of a distractor in pursuit of the response goal [38–41]. In particular, Tipper and colleagues’ population coding model contends that the top-down inhibition of exogenous distractor-related activity in the saccade map of the SC biases the mean vector of saccade-related activity in a direction contralateral to the distractor. Thus, our results may relate to a spatial bias wherein the programmed amplitude of a prosaccade moves further away from the location of a remote distractor to avoid capture of task-irrelevant visual information.

### Antisaccade RTs: Planning costs are independent of a distractor’s spatial location

Antisaccade RTs in each distractor condition were longer than the TO condition, and the magnitude of the distractor cost (average of 36 ms) was independent of the distractor’s spatial location. As such, antisaccade RTs do not elicit a RDE commensurate with prosaccades. Moreover, results demonstrate that distractor-related activity at a remote area of the collicular motor map does not selectively inhibit saccade-related motor activity. Instead, our results suggest that distractors influence antisaccade RTs due to the top-down demands of evoking the response-selection rule necessary for decoupling SR spatial relations. In line with this view, previous work has shown that antisaccades—but not prosaccades—adhere to Hick’s law [22, 42, 43]. According to Kveraga and colleagues, prosaccades violate Hick’s law because an *automatic* response-selection process couples the location of a target stimulus with a motor response. Indeed, the fact that humans complete upwards of 100,000 prosaccades in the course of their daily activities [44] highlights the fact that the prosaccade response-selection process efficiently attenuates task-irrelevant visual information [45]. In turn, Kveraga et al. contend that antisaccades adhere to Hick’s law because the location of a target stimulus cannot be automatically mapped onto the direction of an ensuing response. Instead, the decoupled SR relations engender a cost related to the processing of each potential SR alternative. Moreover, electrophysiological evidence from non-human primates has shown that distinct neural ensembles serve the visual selection of a target stimulus and the selection of an appropriate antisaccade endpoint. For example, Sato and Schall [46] recorded single-cell activity from the frontal eye fields (FEF) of macaques during the planning of pro- and antisaccades to a target stimulus presented within an array of three distractors. Results showed that pro- and antisaccade RT differences were not linked to stimulus identification within the FEF (i.e., disentangling target from distractor); rather, the increase in antisaccade RT was linked to the onset of FEF activity supporting the selection of the task-rule necessary for decoupling SR relations. Notably, our work adds to previous literature insomuch as it demonstrates that that the spatial location of a distractor does not differentially influence the planning time required to adopt an appropriate antisaccade task-rule.

### Effects of proximal and remote distractors on antisaccade endpoints

Antisaccade amplitudes in the ID and CDD conditions were less than and greater than the TO condition, respectively. In turn, amplitudes in the MD and CPD conditions did not reliably differ from the TO condition. In contrast to our results, Viswanathan and Barton [47] reported that antisaccade amplitudes in a remote—but not proximal—distractor condition were consistent with a global effect. Viswanathan and Barton interpreted their results as evidence for a spatial averaging of motor-related activity serving the response goal and distractor location on a common short-range motor map in the intermediate SC. Notably, however, our results do not support a global effect for remote distractors. Indeed, had the present results demonstrated a global effect then the CPD condition would have produced shorter amplitudes than the TO condition because the goal-location of the response is proximal to the distractor’s location (see Fig 3). To our knowledge, our work and Viswanathan and Barton’s represents the only studies to have examined distractor-related influences on antisaccade amplitudes. As well, we are unable to identify a between-experiment methodological difference that might explain the discrepant findings. Moreover, Viswanathan and Barton did not systematically report distractor-related antisaccade RT costs; thus, we are unable to contrast RT and amplitude differences between experiments. As such, we propose that a global effect does not represent a reliable property of antisaccade amplitudes.

In accounting for the finding that ID and CDD conditions produced amplitudes that were distinct from TO trials we note that antisaccade sensorimotor transformations are mediated via a relative visual percept [18–20, 48]. Moreover, the antisaccade visual percept is governed by a strategy of perceptual averaging such that the visual properties of a target are encoded relative to the properties of other stimuli (i.e., distractors) *within* a stimulus-set [49, 50]. Thus, we propose that antisaccade amplitudes are based on a statistical summary of the visual location of the target and distractor. Indeed, for the ID condition the proximity between target and distractor would render a statistical summary and associated visual percept that leads to an increased hypometria. In turn, because the target and distractor in the CDD condition are in opposite visual fields, but have equal eccentricities, a statistical summary would render a more accurate target percept and therefore serve to reduce hypometria. Of course, we emphasize that our perceptual averaging proposal is distinct from the global effect as the former represents a statistical summary of the sensory-related properties contained within a stimulus set [49, 50], whereas the latter is attributed to the weighted average of motor-related activity [8].

## Conclusions

Antisaccades showed a distractor-related increase in RT that was independent of the distractor’s spatial location. Such a finding suggests that stimulus-related activity associated with the distractor at a remote location from the intended saccade goal does not attenuate the motor-related properties of saccade-related neurons serving the antisaccade response. Instead, we propose that distractor-related antisaccade RT costs reflect uncertainty associated with the top-down evocation of the task-rule necessary to decouple SR relations.

## References

[1] Wurtz RH, Albano JE. Visual-motor function of primate superior colliculus. Annu Rev Neurosci. 1980;3: 189–226. 6774653 10.1146/annurev.ne.03.030180.001201

[2] Walker R, Deubel H, Schneider WX, Findlay JM. Effect of remote distractors on saccade programming: Evidence for an extended fixation zone. J Neurophysiol. 1997;78: 1108–1119. 9307138 10.1152/jn.1997.78.2.1108

[3] Lévy-Schoen A. Détermination et latence de la réponse oculomotrice à deux stimulus simultanés ou successifs selon leur excentricité relative. L’An**é**e Psychol. 1969; 69: 373–392.

[4] Coren S, Hoenig P. Effect of non-target stimuli upon length of voluntary saccades. Percept Mot Skills. 1972;34: 499–508. 5063190 10.2466/pms.1972.34.2.499

[5] Deubel H, Wolf W, Hauske G. The evaluation of the oculomotor error signal In: Gale AG, Johnson F, editors, Theoretical and applied aspects of eye movement research. Amsterdam: North Holland; 1984 pp. 55–61.

[6] Findlay JM. Global visual processing for saccadic eye movements. Vision Res. 1982;22: 1033–1045. 7135840 10.1016/0042-6989(82)90040-2

[7] Van der Stigchel S, Nijboer TCW. The global effect: what determines where the eyes land? J Eye Mov Res. 2011;4: 1–13. 21603125

[8] Godijn R, Theeuwes J. Programming of endogenous and exogenous saccades: Evidence for a competitive integration model. J Exp Psychol Hum Percept Perform. 2002;28: 1039–1054. 12421054 10.1037//0096-1523.28.5.1039

[9] Trappenberg TP, Dorris MC, Munoz DP, Klein RM. A model of saccade initiation based on the competitive integration of exogenous and endogenous signals in the superior colliculus. J Cogn Neurosci. 2001;13: 256–271. 11244550 10.1162/089892901564306

[10] Dorris MC, Oliver E, Munoz DP. Competitive integration of visual and preparatory signals in the superior colliculus during saccadic programming. J Neurosci. 2007;27: 5053–5062. 17494691 10.1523/JNEUROSCI.4212-06.2007 PMC6672386

[11] Takahashi M, Sugiuchi Y, Izawa Y, Shinoda Y. Commissural excitation and inhibition by the superior colliculus in tectoreticular neurons projecting to omnipause neuron and inhibitory burst neuron regions. J Neurophysiol. 2005;94: 1707–1726. 16105954 10.1152/jn.00347.2005

[12] Findlay JM, Walker R. A model of saccade generation based on parallel processing and competitive inhibition. Behav Brain Sci. 1999;22: 661–721. 11301526 10.1017/s0140525x99002150

[13] Van Gisbergen JA, Van Opstal AJ, Tax AAM. Collicular ensemble coding of saccades based on vector summation. Neuroscience. 1987;21: 541–555. 3614643 10.1016/0306-4522(87)90140-0

[14] Van Opstal AJ, Van Gisbergen JA. A nonlinear model for collicular spatial interactions underlying the metrical properties of electrically elicited saccades. Biol Cybern. 1989;60: 171–183. 2923922 10.1007/BF00207285

[15] Hallett PE. Primary and secondary saccades to goals defined by instructions. Vision Res. 1978;18: 1279–1296. 726270 10.1016/0042-6989(78)90218-3

[16] Hallett PE, Adams BD. The predictability of saccadic latency in a novel voluntary oculomotor task. Vision Res. 1980;20: 329–339. 7414965 10.1016/0042-6989(80)90019-x

[17] Fischer B, Weber H. Characteristics of “anti” saccades in man. Exp Brain Res. 1992;89: 415–424. 1623983 10.1007/BF00228257

[18] Dafoe JM, Armstrong IT, Munoz DP. The influence of stimulus direction and eccentricity on pro- and anti-saccades in humans. Exp Brain Res. 2007;179: 563–570. 17171535 10.1007/s00221-006-0817-8

[19] Krappmann P, Everling S, Flohr H. Accuracy of visually and memory-guided antisaccades in man. Vision Res. 1998;38: 2979–2985. 9797993 10.1016/s0042-6989(98)00101-1

[20] Heath M, Weiler J, Marriott K, Welsh T. Vector inversion diminishes the online control of antisaccades. Exp Brain Res. 2011;209: 117–127. 10.1007/s00221-010-2525-7 21210087

[21] Munoz DP, Everling S. Look away: The anti-saccade task and the voluntary control of eye movement. Nat Rev Neurosci. 2004;5: 218–228. 14976521 10.1038/nrn1345

[22] Kveraga K, Boucher L, Hughes HC. Saccades operate in violation of Hick’s Law. Exp Brain Res. 2002;146: 307–314. 12232687 10.1007/s00221-002-1168-8

[23] Hick WE. On the rate of gain of information. Q J Exp Psychol. 1952;4: 11–26.

[24] Brainard DH. The Psychophysics Toolbox. Spat Vis. 1997;10: 433–436. 9176952

[25] Olk B, Kingstone A. Why are antisaccades slower than prosaccades? A novel finding using a new paradigm. Neuroreport. 2003;14(1): 151–155. 12544848 10.1097/00001756-200301200-00028

[26] DeSimone JC, Weiler J, Aber GS, Heath M. The unidirectional prosaccade switch cost: Correct and error antisaccades differentially influence the planning times for subsequent prosaccades. Vision Res. 2014;96: 17–24. 10.1016/j.visres.2013.12.005 24412739

[27] Weiler J, Heath M. Oculomotor task switching: alternating from a nonstandard to a standard response yields the unidirectional prosaccade switch-cost. J Neurophysiol. 2014;112: 2176–2184. 10.1152/jn.00352.2014 25122700

[28] Loftus GR, Masson ME. Using confidence intervals in within-subjects designs. Psychon Bull Rev. 1994;1: 476–490. 10.3758/BF03210951 24203555

[29] Cumming G. Understanding the new statistics: Effect sizes, confidence intervals, and meta-analysis New York: Routledge; 2011.

[30] Cumming G. The new statistics: Why and how. Psychol Sci. 2013;25: 7–29. 10.1177/0956797613504966 24220629

[31] Kowler E, Blaser E. The accuracy and precision of saccades to small and large targets. Vision Res. 1995;35: 1741–1754. 7660582 10.1016/0042-6989(94)00255-k

[32] Casteau S, Vitu F. On the effect of remote and proximal distractors on saccadic behavior: A challenge to the neural-field models. J Vis. 2012;12(14): 1–33. 10.1167/12.12.14 23184233

[33] Dorris MC, Munoz DP. Saccadic probability influences motor preparation signals and time to saccadic initiation. J Neurosci. 1998;18: 7015–7026. 9712670 10.1523/JNEUROSCI.18-17-07015.1998 PMC6792986

[34] Ottes FP, Van Gisbergen JA, Eggermont JJ. Latency dependence of colour-based target vs nontarget discrimination by the saccadic system. Vision Res. 1985;25: 849–862. 4024483 10.1016/0042-6989(85)90193-2

[35] Gaveau V, Martin O, Prablanc C, Pelisson D, Urquizar C, Desmurget M. On-line modification of saccadic eye movements by retinal signals. Neuroreport. 2003;14: 875–878. 12858051 10.1097/00001756-200305060-00020

[36] West GL, Welsh TN, Pratt J. Saccadic trajectories receive online correction: Evidence for a feedback-based system of oculomotor control. J Mot Behav. 2009;41: 117–127. 10.3200/JMBR.41.2.117-127 19201682

[37] Benson V. A comparison of bilateral versus unilateral target and distractor presentation in the remote distractor paradigm. Exp Psychol. 2008;55(5): 334–341. 10.1027/1618-3169.55.5.334 25116301

[38] Doyle M, Walker R. Curved saccade trajectories: Voluntary and reflexive saccades curve away from irrelevant distractors. Exp Brain Res. 2001;139: 333–344. 11545472 10.1007/s002210100742

[39] Tipper SP, Howard LA, Houghton G. Action-based mechanisms of attention. Philos Trans R Soc Lond B Biol Sci. 2000;353: 1385–1393. 9770231 10.1098/rstb.1998.0292 PMC1692337

[40] Tipper SP, Howard LA, Paul MA. Reaching affects saccade trajectories. Exp Brain Res. 2001;136: 241–249. 11206286 10.1007/s002210000577

[41] Walker R, McSorley E. The influence of distractors on saccade-target selection: Saccade trajectory effects. J Eye Mov Res. 2008;2: 7:1–13.

[42] Kloft L, Reuter B, Viswanathan J, Kathmann N, Barton JJS. Response selection in prosaccades, antisaccades, and other volitional saccades. Exp Brain Res. 2012;222: 245–353. 10.1007/s00221-012-3218-1 22910901

[43] Lawrence BM. An anti-Hick’s effect for exogenous, but not endogenous, saccadic eye movements. Exp Brain Res. 2010;204: 115–188. 10.1007/s00221-010-2301-8 20521032

[44] Irwin DE, Thomas LE. The effect of saccades on number processing. Percept Psychophys. 2007;69: 450–458. 17672432 10.3758/bf03193765

[45] Pashler HE. The psychology of attention Cambridge, MA: MIT Press; 1998.

[46] Sato TS, Schall JD. Effects of stimulus-response compatibility on neural selection in frontal eye field. Neuron. 2003;38: 637–648. 12765614 10.1016/s0896-6273(03)00237-x

[47] Viswanathan J, Barton JJS. The global effect for antisaccades. Exp Brain Res. 2013; 225(2): 247–259. 10.1007/s00221-012-3366-3 23254509

[48] Evdokimidis I, Tsekou H, Smyrnis N. The mirror antisaccade task: direction-amplitude interaction and spatial accuracy characteristics. Exp Brain Res. 2006;174: 304–311. 16636790 10.1007/s00221-006-0462-2

[49] Gillen C, Heath M. Target frequency influences antisaccade endpoint bias: Evidence for perceptual averaging. Vision Res. 2014;105: 151–158. 25449162 10.1016/j.visres.2014.10.010

[50] Gillen C, Heath M. Perceptual averaging governs antisaccade endpoint bias. Exp Brain Res. 2014;232: 3201–3210. 10.1007/s00221-014-4010-1 24935477

